# Influence of Seropositivity against Adenovirus-36 on the Risk of Obesity and Insulin Resistance in the Child Population of Southern Chile

**DOI:** 10.3390/v16060995

**Published:** 2024-06-20

**Authors:** Roberto Brito, Jorge Sapunar, Nicolás Aguilar-Farías, Juan Navarro-Riquelme, Monica Pavez, Mario Hiroyuki Hirata, Alvaro Cerda

**Affiliations:** 1 Center of Excellence in Translational Medicine CEMT-BIOREN, Universidad de La Frontera, Temuco 4810296, Chile; r.brito01@ufromail.cl (R.B.); jorge.sapunar@ufrontera.cl (J.S.); monica.pavez@ufrontera.cl (M.P.); 2 Department of Internal Medicine, Universidad de La Frontera, Temuco 4781176, Chile; 3 Department of Physical Education, Sports and Recreation, Universidad de La Frontera, Temuco 4811230, Chile; nicolas.aguilar@ufrontera.cl; 4 Department of Pediatrics and Children’s Surgery, Universidad de La Frontera, Temuco 4781176, Chile; dr.janavarro@gmail.com; 5 School of Pharmaceutical Sciences, University of Sao Paulo, Sao Paulo 05508-060, Brazil; mhhirata@usp.br; 6 Department of Basic Sciences, Universidad de La Frontera, Temuco 4811230, Chile

**Keywords:** Adenovirus-36, childhood obesity, insulin resistance

## Abstract

**Background:** Previous infection with Adenovirus-36 (HAdv-D36) has been associated with adipogenesis and glycemic regulation in cell culture and animal models. In humans, HAdv-D36 antibodies correlate with increased obesity risk yet paradoxically enhance glycemic control across various demographics. This study assesses the association of HAdv-D36 seropositivity with obesity, lipid, and glycemic profiles among school-aged children. **Methods**: We evaluated 208 children aged 9–13, categorized by BMI z-scores into normal weight (−1 to +1), overweight (+1 to +2), and obese (>+3). Assessments included anthropometry, Tanner stage for pubertal development, and biochemical tests (relating to lipids, glucose, and insulin), alongside HAdv-D36 seropositivity checked via ELISA. Insulin resistance was gauged using Chilean pediatric criteria. **Results**: The cohort displayed a high prevalence of overweight/obesity. HAdv-D36 seropositivity was 5.4%, showing no correlation with nutritional status. Additionally, no link between HAdv-D36 seropositivity and lipid levels was observed. Notably, insulin levels and HOMA-RI were significantly lower in HAdv-D36 positive children (*p* < 0.001). No cases of insulin resistance were reported in the HAdv-D36 (+) group in our population. **Conclusions**: HAdv-D36 seropositivity appears to decrease insulin secretion and resistance, aligning with earlier findings. However, no association with obesity development was found in the child population of southern Chile.

## 1. Introduction

Obesity is a serious public health issue and is a risk factor for the development of cardiovascular diseases (CVDs), type 2 diabetes (T2DM), and cancer, which are the leading causes of death in developed countries [1]. Although these effects manifest during adulthood, the risk factors that promote them can be detected at early ages [2]. Childhood obesity has become a global emerging issue, even in countries where malnutrition used to be the primary concern. It is known that 80% of children with overweight or obesity go on to become adults with obesity [3]. In this context, Chile has a high level of childhood obesity, ranking as the South American country with the highest prevalence of childhood and adolescent obesity, with 27.3% of its youth population being overweight and 31.0% presenting with obesity [4].

Childhood obesity is defined using the BMI (Body Mass Index) z-score, which involves normalizing BMI data adjusted for biological age and gender, enabling the comparison of heterogeneous groups. The etiology of obesity is complex, resulting from the interaction of genetic and environmental factors such as a sedentary lifestyle, inadequate diet, and sociocultural and psychological factors [5]. One of the least-studied environmental factors is infectobesity, which refers to the contribution of infectious agents in the development of obesity through their role in stimulating the proliferation and differentiation of adipose cells [6].

Adenovirus-36 (HAdv-D36) has been shown to stimulate adipogenic differentiation and facilitate glucose transport in experimental cell culture models of murine preadipocytes and human cells [7]. In animal experimental models, HAdv-D36 has been shown to induce weight gain and obesity, promote an increase in body fat, and lead to an improvement in glycemic control [8]. Human adenoviruses are double-stranded DNA viruses that are implicated in infections of the upper respiratory tract and gastrointestinal tract. These infections are common in children due to their lack of acquired immunity [9]. In humans, HAdv-D36 produces a self-limiting infection whose seropositivity has been correlated with a higher risk of obesity in adults in various populations. The most recent meta-analysis that evaluated cross-sectional and case–control studies in distinct populations observed a higher risk of obesity in seropositive individuals, with a combined Odds Ratio (OR) for the studies considered of 1.84 (95% CI: 1.39–2.43) [10]. In the Chilean adult population, a higher prevalence of antibodies against HAdv-D36 has previously been reported in individuals with obesity compared to normal weight controls, suggesting that people previously infected with HAdv-D36 have a higher risk of obesity in our population (OR: 2.67; 95% CI: 1.58–4.51, *p* < 0.001) [11].

On the other hand, the correlation between seropositivity against HAdv-D36 and childhood obesity has been described in different populations; however, there is no information of this nature for the Chilean child population. The present work evaluated the influence of seropositivity against HAdv-D36 on the risk of obesity, lipid, and glycemic profiles in a population of school-age children from southern Chile.

## 2. Materials and Methods

### 2.1. Study Population

Our cross-sectional study included schoolchildren from the 4th to 6th year of basic education, corresponding to ages 9 to 13, from the commune of Carahue, Araucanía Region, Chile. The study subjects were selected from six schools through multi-stage random sampling according to the school, geographical location, and enrollment size. Both urban and rural populations were included. The study protocol was approved by the scientific ethics committee of the Universidad de La Frontera (Protocols number 026/15 and 148/21). The students agreed to participate by signing their informed assent, which was endorsed by their parent or guardian by signing an informed consent form.

### 2.2. Clinical Evaluation, Anthropometric Measurements, and Nutritional Status

Biodemographic data were collected and a pediatric clinical evaluation including an evaluation of pubertal development and anthropometry was performed. Participants were categorized according to pubertal development (Tanner stages). Weight and height were measured by a scale and stadiometer, while nutritional status was determined according to the percentile criteria of the American Center for Disease Control (CDC) by calculating the BMI z-score normalized by biological age. The waist and hip circumference were measured with a non-extensible measuring tape at the level of the navel and trochanters, respectively, and were used to calculate the waist/hip ratio (WHR). The cervical perimeter and arm circumference were also obtained). Systolic and diastolic blood pressures (SBP and DBP) were measured with a pediatric sphygmomanometer, and the average of two consecutive measurements was recorded. Individuals with SBP and DBP values higher than the 90th percentile for their age and sex according to the recommendation of the National High Blood Pressure Education Program (NHBPEP) [12] were considered hypertensive. Abdominal obesity was defined by a waist circumference greater than the 90th percentile according to age and sex.

### 2.3. Biochemical Determinations, Definition of Insulin Resistance and Dyslipidemias

Fasting blood samples (10 to 12 h) obtained by venipuncture were used to perform biochemical determinations and seropositivity against HAdv-D36. The plasma concentration of glucose, total cholesterol (TC), HDL cholesterol (HDL-c), and triglycerides (TG) was determined by enzymatic–colorimetric methods. The concentration of VLDL cholesterol (VLDL-c) was calculated as one-fifth of the concentration of triglycerides (TG/5), the concentration of LDL-c was calculated using the Friedewald Formula, and the concentration of non-HDL cholesterol (noHDL-c) was calculated as the difference between total cholesterol and HDL-c. The insulin concentration was determined by chemiluminescence. Biochemical and hormonal analyses were performed on Roche-Cobas 331 and 411 instruments (Roche Diagnostics, Basel, Switzerland). Glucose and insulin values were used to calculate the Homeostatic model assessment–insulin resistance (HOMA-IR) index. To define the presence of insulin resistance (IR), the HOMA-IR was used using the criteria for the Chilean pediatric population proposed by Barja et al. [13], considering the Tanner stage and the sex of the individuals. The presence of dyslipidemia according to criteria for the pediatric population was defined as previously described in the work of our group [14]. It was possible to obtain 205 blood samples from the participants.

### 2.4. Seropositivity against HAdv-D36

Determination of the presence of Anti-HAdv-D36 antibodies was performed using the Adenovirus-36 Antibody (AdV36-Ab) ELISA Kit (MyBiosource #MBS9310682, San Diego, CA, USA, EU) according to the manufacturer’s instructions. Serum samples were processed in duplicate and classified as HAdv-D36 seronegative or seropositive according to the recommended absorbance cut-off value (0.15 OD). All samples with absorbances in an inconclusive range (OD 0.14–0.16) were reprocessed to confirm the result. Finally, 15% of the samples were randomly selected to repeat the analysis, obtaining 100% coincidence.

### 2.5. Statistical Analysis

The results were analyzed using Minitab v.17 statistical software (Minitab Inc., State College, PA, USA, EU) and Graphpad Prism v.8 (GraphPad Software, BO, EU). A normality analysis was performed for each continuous variable using the Kolmogorov–Smirnov test and descriptive statistics of the variables were performed. Chi-square tests and Fisher’s test were used to compare categorical variables. The comparison between groups was performed by *t*-test or ANOVA (one-way) followed by Tukey’s test for variables with normal distribution. Variables with nonparametric distribution were compared by the Mann–Whitney or Kruskal–Wallis tests followed by a Dunn test. Statistical significance was set at *p* < 0.05.

## 3. Results

The biodemographic, clinical, anthropometric, and biochemical parameters and the frequency of anti-HAdv-D36 antibodies of the study population according to nutritional status are presented in Table 1. The male sex predominated in the group of individuals with obesity (*p* = 0.029). The presence of dyslipidemia was 38% and the IR was 19% in the total group, with conditions being significantly more frequent in the group of individuals with overweight and group of individuals with obesity (*p* < 0.05). SBP was higher in the group with obesity compared to the normal weight group (*p* < 0.001), while no difference was observed for DBP. All anthropometric parameters gradually increased among overweight and obese groups (*p* < 0.001) compared to the normal weight group.

Individuals with obesity had higher concentrations of triglycerides, non-HDL cholesterol, and VLDL, while HDL cholesterol was lower in this group (*p* < 0.005). Although there were no differences in glycemic values according to nutritional status, elevated insulin and HOMA-IR values were observed in the group of individuals subjects with obesity compared to individuals with normal weight (*p* < 0.001).

Overall, 5.4% of the study population displayed HAdv-D36 seropositivity. The biodemographic, clinical, anthropometric, and biochemical parameters of the study population according to the presence of the HAdv-D36 antibody are presented in Table 2.

The HAdv-D36(+) group showed a trend toward lower age values, which did not reach statistical significance (*p* = 0.054). Abdominal obesity was only present in the seronegative group, which agrees with the results observed for waist circumference, cervical perimeter, and arm circumference, which were significantly higher in the seronegative group.

**Figure 1:**
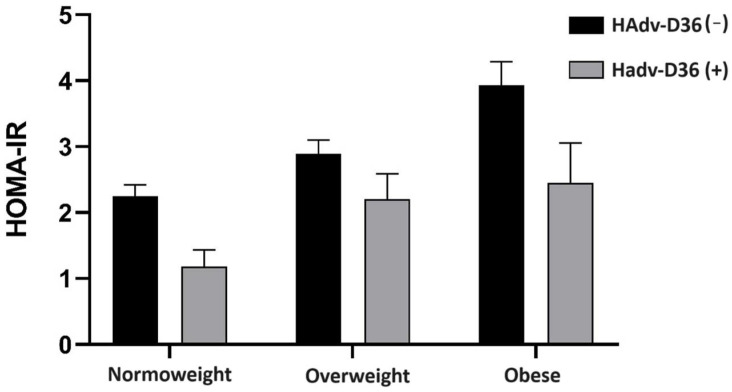
Influence of seropositivity against HAdv-D36 on HOMA-IR according to nutritional status. Bars represent mean and standard error of the mean. HAdv-D36 (−) and HAdv-D36 (+) denote negative serology and positive serology against HAdv-D36, respectively. HOMA-IR: insulin resistance index according to the homeostatic assessment model.

No significant differences were observed between the seropositive and seronegative groups in the biochemical parameters of the lipid profile and glycemia (*p* > 0.05). Interestingly, IR was significantly less frequent in the HAdv-D36 seropositive group (*p* = 0.029), given that in this group with previous infection (HAdv-D36 (+)), no subjects with IR were reported. Consequently, insulin concentration (*p* = 0.003) and HOMA-IR (0.001) were significantly lower in the seropositive group. As seen in Figure 1, the trend of lower values of the HOMA-IR insulin resistance index in HAdv-D36 (+) subjects is maintained regardless of nutritional status.

## 4. Discussion

In this study, we found that seropositivity to HAdv-D36 was not related to nutritional status; however, individuals previously infected by the virus had lower insulin levels and a lower risk of insulin resistance. It is likely that the lack of relationship with nutritional status could be due to several factors, including the low seropositivity found in this population, as well as the high level of childhood overweight and obesity in the study population, which could have masked the effect of the virus.

Infectobesity, or obesity of infectious origin, and particularly that related to HAdv-D36, has aroused interest in recent years as a new environmental risk factor for the development of obesity. HADv-D36 has been shown to promote adipogenic differentiation. Adipose tissue is mainly composed of adipocytes, which come from mesenchymal stem cells [15]. Adipogenic differentiation is a sequential process that requires various transcription factors, CAAT-binding enhancer protein (C/EBP), and peroxisome proliferator-activated receptor gamma (PPARγ) genes, which are considered “master switches” of the adipogenic process [16]. Research on murine and human cell lines has demonstrated that HAdv-D36 infection causes an increase in adipogenic differentiation and an increase in glycerol-3-phosphate dehydrogenase (GPDH) [17].

This study has some limitations, such as the method used to screen HAdv-D36 antibodies. The serum neutralization assay (SNA) is the gold standard for specifically detecting antibodies capable of neutralizing HAdv-D36. Although an enzyme immunoassay provides a quicker and more objective determination, it could be non-specific, with some false-positive results. Nevertheless, reported cross-sectional and case–control studies have widely used both ELISA and SNA [18]. Indeed, as the ELISA method is related to false higher seroprevalence results in a sample population, it is unlikely that the low prevalence observed here was affected by methodological specificity. Moreover, although our results are not confirmed by SNA, exhaustive quality control was performed to confirm the results of the serology. On the other hand, the low prevalence of Ad-36+ in our study could affect the power of the statistical tests due to the small sample size.

The correlation between HAdv-D36 seropositivity and childhood obesity has been described in different populations. In a study involving Turkish children, seropositivity was 27.1% in the group of individuals with obesity and 6% in the group of individuals without obesity, while in adults it was 17.5% in the group of individuals with obesity and 4% in the group of individuals without obesity, with a significant difference in both groups, suggesting that the effect of the virus could be greater in the child population [18]. In Korean schoolchildren, seropositivity in the group of individuals with obesity was 28.57% compared to 13.56% in the group of individuals without obesity [19]. In Sweden, seropositivity was associated with childhood obesity and severe obesity in women, which was found to be 1.5–2 times higher in the latter study population than in the normal weight/overweight/obese group [20]. In our population, seropositivity in the obese group was 4.4% and 5.8% in the non-obese group, without statistical significance. This may be due to the low number of seropositive individuals, as well as the high impact of other obesogenic factors. In other Latin American populations, the prevalence of seropositive infants was higher: in the Brazilian population it was 15.8% [21] and in the Mexican population it was 73.8% [22]. Due to the above findings, it is not possible to ensure that HAdv-D36 does not have an impact on obesity in our population. On the other hand, an important limitation of the present study is that the sample was selected from a single commune, and our results may not be representative of the prevalence of previous infection with HAdv-D36 for our population.

In the adult population, a paradoxical reduction in serum lipids has been reported in humans carrying anti-HAdv-D36 antibodies. Seropositivity has been correlated with lower cholesterol and triglyceride levels in participants with obesity in the United States [23]. In the Korean population, lower triglyceride levels were observed in individuals with normal weight, overweight, and obesity [24]. This association has not been found in the Han Chinese population [25]. A study in a Turkish child population observed that HAdv-D36 seropositive children had lower levels of adiponectin and higher levels of IL-6; however, there was no significant difference with regard to serum lipids [26]. In the US population, it was observed that seropositive children had higher levels of IL-6 and TNFα, so more evidence is required to establish whether there is an influence of viral infection on lipid metabolism and the risk of dyslipidemia.

There is a significant difference in the frequency of seropositivity to HAdv-D36 in our results compared to previous reports in the Chilean adult population [11]. This disparity could be influenced by some epidemiological differences in the sample population from both studies. Whereas people from all over southern Chile submitted to an elective bariatric surgery were selected in the previous published study evaluating adult population, the present work on child population was performed by recruiting subjects from a unique geographical area with a high percentage of rurality.

The most recent meta-analysis published by Fernandes and collaborators in 2021 discussed the available literature, including the effect on the pediatric population. In this work, the disparity between seropositivity against HAdv-D36 between different populations was observed: the average was 28.9%, with a range between 7.5% and 73.9% [27]. Although it was observed in most studies that previous infection is related to an increased risk of obesity, this relationship was not observed in populations with a low seroprevalence [27].

Few studies have related glycemic control to HAdv-D36 infection, even in adults. In Swiss adults, a lower frequency of T2D and better insulin sensitivity have been reported with seropositivity, especially in women [20]. The authors described that normal glucose tolerance was more common in the seropositive group than in the seronegative group. Similarly, a study that compared indices of adiposity and glycemic control in adults previously infected with HAdv-D36 and adults who were uninfected showed greater adiposity and an attenuation of the deterioration of glycemic control in the seropositive group [20].

In the child population, the evidence is more limited. HAdv-D36 seropositivity has been associated with an increase in serum lipids and leptin and a decrease in adiponectin [26]. In our study, HAdv-D36 seropositivity was associated with lower insulin, lower HOMA-IR, and a lower risk of IR. However, there was no association between seropositivity and glycemia. The relationship of seropositivity against HAdv-D36 on glycemia in children was evaluated in Brazilian and Mexican populations, and again, no relationship was observed [21,22]. However, it should be noted that glycemic alterations are infrequently observed during childhood, as they occur once the compensatory mechanisms of insulin sensitivity and secretion are not sufficient to maintain a euglycemic state [28].

## Figures and Tables

**Table 1:** Anthropometric, clinical, metabolic and serological data of normal weight group, overweight group, and obese group.

Variables	Total Group (208)	Normal Weight Group (60)	Overweight Group (79)	Obese Group (69)	*p*-Value
Clinical and biodemographic data					
Age, years	10.4 ± 1.0	10.3 ± 1.0	10.4 ± 1.0	10.4 ± 1.1	0.647
Tanner Stage [1–2/3–5], %	66.2 (137) /33.8 (70)	60.0 (36) /40.0 (24)	62.8 (49) /37.2 (29)	75.0 (51) /25.0 (17)	0.144
Sex [M/F], %	48.3/51.7 (101/107)	35.0/65.0 (21/39)	50.6/49.4 (40/39)	58.0/42.0 (40/29)	0.029
Dyslipidemia %	38.3 (78)	24.6 (14)	34.2 (27)	54.4 (37)	0.002
Insulin Resistance, %	19 (39)	5 (3)	15 (12)	35 (24)	<0.001
SBP, mmHg DBP, mmHg	109.6 ± 18.6 61.5 ± 16.7	105.3 ± 18.4 ^a^ 63.9 ± 17.8	107.6 ± 18.3 57.8 ± 13.8 ^a.b^	115.7± 18.1 ^b^ 64.4 ± 18.3	<0.001 0.190
Hypertension %	35.5 (55)	28.8 (13)	28.8 (17)	49.0% (25)	0.050
Anthropometric Parameters					
BMI z-score	1.50 ± 0.67	0.35 ± 0.55 ^a^	1.42 ± 0.28 ^b^	2.58 ± 0.40 ^c^	<0.001
Body fat, %	28.4± 8.7	21.7± 6.8 ^a^	27.3 ± 5.7 ^b^	36.4 ± 7.1 ^c^	<0.001
Biochemical Parameters					
Total Cholesterol, mg/dL	154 ± 29	150 ± 32	154 ± 27	157 ± 28	0.392
Triglycerides, mg/dL	99 ± 68	80 ± 31 ^a^	90 ± 37 ^a^	127 ± 102 ^b^	<0.001
LDL cholesterol, mg/dL	82 ± 24	79 ± 25	85 ± 23	87 ± 24	0.171
HDL cholesterol, mg/dL	51 ± 13	54 ± 14 ^a^	52 ± 12 ^a^	46 ± 13 ^b^	<0.001
Non-HDL cholesterol, mg/dL	103 ± 30	97 ± 27 ^a^	103 ± 25 ^a.b^	112 ± 30 ^b^	0.003
VLDL cholesterol, mg/dL	20 ± 14	16 ± 6 ^a^	18 ± 8 ^a^	26 ± 21 ^b^	<0.001
Glucose, mg/dL	85 ± 7	85 ± 6	85 ± 6	86 ± 9	0.844
Insulin, μU/Ml	14.1 ± 9.4	10.1 ± 5.5 ^a^	13.5 ± 8.2 ^b^	18.0 ± 11.6 ^c^	<0.001
HOMA-IR	3.00 ± 2.58	2.15 ± 1.25 ^a^	2.86 ± 1.82 ^a^	3.86 ± 2.81 ^b^	<0.001
Serology					
Anti-HAdv-D36, %	5.4% (11)	8.8% (5)	3.8% (3)	4.4% (3)	0.089

The number of individuals is indicated in parentheses. Categorical data are presented as a per-centage and were compared using the chi-square test. Continuous variables are shown as mean ± standard deviation and were compared using ANOVA followed by Tukey’s method or Kruskal–Wallis followed by Dunn’s method for parametric and nonparametric variables, respectively. The children were grouped according to their pubertal development into Tanner stage 1–2 or 3–5. SBP: systolic blood pressure; DBP: diastolic blood pressure; BMI: body mass index; LDL: low-density lipoprotein; HDL: high-density lipoprotein; VLDL: very low-density lipoprotein; HOMA-IR: re-sistance index insulin according to the homeostatic evaluation model; Anti-HAdv-D36: positive serology against HAdv-D36 a, b, c: different letters on the same line represent significant differences.

**Table 2:** Main characteristics of the study population according to the presence of antibodies against HAdv-D36.

Variables	HAdv-D36 (+) (11)	HAdv-D36 (−) (194)	*p*-Value
Clinical and biodemographic data			
Age, years	9.7 ± 1.01	10.4 ± 1.02	0.054
Tanner Stage [1–2/3–5], %	72.7 (8)/27.3 (3)	66.2 (127)/33.9 (65)	0.647
Sex [M/F], %	45.5 (5)/55.6 (6)	49.5 (96)/50.5 (98)	0.795
Dyslipidemia %	36.4 (4)	38.2 (74)	0.906
SBP, mmHg	107.7 ± 15.5	109.4 ± 18.7	0.864
DBP, mmHg	67.0 ± 18.4	61.2 ± 16.7	0.642
Insulin Resistance, %	0 (0)	20.1 (39)	0.029
Anthropometric Parameters			
BMI z-score	1.12 ± 0.90	1.53 ± 0.97	0.174
Body fat, %	24.0 ± 7.5	28.8 ± 8.8	0.084
Biochemical Parameters			
Total cholesterol, mg/dL	153.3 ± 17	154 ± 29	0.895
Triglycerides, mg/dL	99 ± 54	100 ± 69	0.953
LDL Cholesterol, mg/dL	88 ± 13	84 ± 24	0.346
HDL Cholesterol, mg/dL	46 ± 8	51 ± 14	0.063
Non-HDL cholesterol, mg/Dl	107 ± 13	103 ± 29	0.347
VLDL cholesterol, mg/dL	21 ± 12	20 ± 14	0.833
Glucose, mg/dL	83 ± 5	85 ± 7	0.134
Insulin, μU/mL	8.9 ± 4.7	14.4 ± 9.5	0.003
HOMA-IR	1.81 ± 0.86	3.07 ± 2.21	0.001

Note: The number of individuals is in parentheses. Individuals were grouped according to seropositivity against HAdv-D36 (HAdv-D36 (−) and HAdv-D36 (+) represent negative and positive serology, respectively). Categorical data are presented as a percentage and were compared using the chi-square test. Continuous variables are shown as mean ± standard deviation and were compared using a *t*-test or Mann–Whitney U test for parametric and nonparametric variables, respectively. SBP: systolic blood pressure; DBP: diastolic blood pressure; BMI: body mass index; LDL: low-density lipoprotein; HDL: high-density lipoprotein; VLDL: very-low-density lipoprotein, HOMA-IR: resistance index insulin according to the homeostatic model.

## Data Availability

The data presented in the present study are available upon request from the corresponding author. The data are not publicly available due to privacy and ethical restrictions.
